# A novel approach to frontline health worker support: a case study in increasing social power among private, fee-for-service birthing attendants in rural Bangladesh

**DOI:** 10.1186/s12960-022-00773-6

**Published:** 2023-02-07

**Authors:** Dora Curry, Md. Ahsanul Islam, Bidhan Krishna Sarker, Anne Laterra, Ikhtiar Khandaker

**Affiliations:** 1 CARE (formerly for Curry, Islam, and Laterra; current for Khandaker), Atlanta, USA; 2 University of Georgia, Athens, Georgia; 3 International Centre for Diarrhoeal Disease Research, Bangladesh (formerly CARE-Bangladesh), Dhaka, Bangladesh

**Keywords:** Frontline health workers, Agency, Skilled birthing attendants, Health workforce

## Abstract

**Background:**

Expanding the health workforce to increase the availability of skilled birth attendants (SBAs) presents an opportunity to expand the power and well-being of frontline health workers. The role of the SBA holds enormous potential to transform the relationship between women, birthing caregivers, and the broader health care delivery system. This paper will present a novel approach to the community-based skilled birth attendant (SBA) role, the Skilled Health Entrepreneur (SHE) program implemented in rural Sylhet District, Bangladesh.

**Case presentation:**

The SHE model developed a public–private approach to developing and supporting a cadre of SBAs. The program focused on economic empowerment, skills building, and formal linkage to the health system for self-employed SBAs among women residents. The SHEs comprise a cadre of frontline health workers in remote, underserved areas with a stable strategy to earn adequate income and are likely to remain in practice in the area. The program design included capacity-building for the SHEs covering traditional techno-managerial training and supervision in programmatic skills and for developing their entrepreneurial skills, professional confidence, and individual decision-making. The program supported women from the community who were social peers of their clients and long-term residents of the community in becoming recognized, respected health workers linked to the public system and securing their livelihood while improving quality and access to maternal health services. This paper will describe the SHE program's design elements to enhance SHE empowerment in the context of discourse on social power and FLHWs.

**Conclusion:**

The SHE model successfully established a private SBA cadre that improved birth outcomes and enhanced their social power and technical skills in challenging settings through the mainstream health system. Strengthening the agency, voice, and well-being of the SHEs has transformative potential. Designing SBA interventions that increase their power in their social context could expand their economic independence and reinforce positive gender and power norms in the community, addressing long-standing issues of poor remuneration, overburdened workloads, and poor retention. Witnessing the introduction of peer or near-peer women with well-respected, well-compensated roles among their neighbors can significantly expand the effectiveness of frontline health workers and offer a model for other women in their own lives.

## Background

The Sustainable Development Goals (SDG) for 2030 target reducing the Maternal Mortality Ratio to 70 maternal deaths per 100,000 live births. Increased availability of skilled birth attendants (SBAs) is well established as one essential ingredient of reducing maternal mortality and is a primary indicator for documenting progress in this area [1–3]. Within a system-wide approach to improving maternal health outcomes, universal availability of skilled birthing care is one critical element of achieving progress on this crucial SDG [5–7]. The WHO Global Strategy for Human Resources for Health: Workforce 2030 calls on countries to increase investment in frontline health workers and explore new ways to optimize health service expertise [8].

Expanding the health workforce to increase the availability of SBAs presents an opportunity. The role of the SBA holds enormous potential to transform the relationship between women, birthing caregivers, and the broader health care delivery system. This paper will focus on the community-based skilled birth attendant (SBA) role and its transformative potential, using a novel approach to SBAs, implemented in rural Sylhet District, Bangladesh, as an illustrative example. The introduction of diverse processes, like this one, to increase the uptake of basic skilled birthing care can play an essential role in improving coverage with skilled birthing attendants. In addition, insights from such new approaches to financing and supporting frontline health workers can contribute to health workforce expansion and quality improvement in health areas beyond safe delivery.

The Skilled Health Entrepreneur (SHE) model developed a public–private approach to developing and training a cadre of SBAs. The program focused on economic empowerment, skills building, and formal linkage to the health system for self-employed SBAs among women residents. This model shifts the view of community-based birth attendants from one of a substandard, stopgap force extender to one of a unique class of skilled providers. The program invests the SHEs with income, autonomy, and external professional recognition. Creating a cadre of providers of similar socioeconomic status and culture to clients enhances the value of the SBA and her services in her clients' eyes.

The Sumanganj District of Bangladesh provides a valuable context to explore these issues in several ways. Not only does the area experience a critical gap in the availability of health care service providers, but a market also exists for fee-for-service health care, as community members are already accustomed to seeking care or unreliable quality from often unskilled private providers due to the gap in the availability of providers in public facilities. In addition, an established cadre of community-based skilled birth attendants already existed, but was underutilized due mainly to inadequate supervision and low community awareness of their capabilities. Finally, women faced barriers to seeking delivery services at facilities due to social norms and religious practices [9].

Other models exist with some similarities. For example, this model is similar to the Shasthya Shebika (SS) approach. The SHE and the SSs are selected from the community, provided training and supervision, provided community-based services, and rely on their activities to earn compensation. The distinctive element of the SHE approach is that the SHEs charge for their services directly on a fee-for-service basis. SSs receive a financial incentive from relatively small mark-ups of resale health-related products provided or subsidized by a sponsoring organization such as an INGO or the MOH. This feature also sets the SHE model apart from similar models in other countries, like kaders’ posyandu in Indonesia or the LiveWell model in Zambia [4].

This paper will first present an overview of factors influencing the uptake of skilled birthing care and then describe the SHE model and its transformational potential. The SHE model comprises a cadre of frontline health workers in remote, underserved areas with a stable strategy to earn adequate income and are likely to remain in practice in the area. They can provide high-quality basic clinical skills and access to higher care. The community and the health system recognize them as legitimate. In addition, they are female, come from the same geographical, and cultural background as their clients, and are closer to their clients' socioeconomic peers than most other health workers.

These features of the SHE model can potentially increase clients' uptake of skilled birthing services and contribute positively to social and gender dynamics. Selecting SBAs from among women within traditionally underrepresented and marginalized communities ensures that they have networks, social connections, capital, and a desire to continue building a life there. Designing SBA interventions that increase their power in their social context could expand their economic independence and reinforce positive gender and power norms in the community, addressing long-standing issues of poor remuneration, overburdened workloads, and poor retention.

These shifts could also enhance the perception of quality and accessibility among clients and contribute more to women’s agency. This model amplifies and gives greater weight to client perception and builds on frontline providers’ and clients’ agency, making it more robust in challenging settings, more acceptable to clients, and more sustainable than other options.

## Methods

This paper is a descriptive exercise depicting a novel intervention in detail. A selective review of relevant literature provides an overview of maternal health strategies to improve skilled birth attendant availability and skill. The literature review included both peer-reviewed publications and "grey" literature. The project description draws on an in-depth desk review of project documentation. The desk review covered the project proposal, routine project reporting covering supportive supervision findings, training materials, activity logs, and internal assessments; midline and end-line reports; and journal articles published on program data. Program monitoring and evaluation data included in the review covered project outputs such as health services delivered, commodities sold, community events conducted, and project outcomes such as the percentage of the coverage area accessing critical maternal and child health services.

## Case presentation

International calls for more significant investment in skilled birthing care underestimate the complexity of women's needs and preferences and providers' needs and preferences [7]. To maximize the impact of such investments, health worker support interventions must offer a specific pathway to address the unique challenges of a range of women's preferences [11]. Women's preference for birthing care that is convenient, respectful, or culturally congruent may overshadow clinical quality, as defined by technical experts, in their care-seeking.

Afulani and Moyer proposed a framework that includes perceived need, accessibility, and quality as three factors affecting the uptake of skilled birthing care [12]. The critical insight their analysis contributes model is the influence of client perception on their decisions about seeking services. Distinguishing between perceived quality and accessibility, on the one hand, and clinical quality and distance to care, on the other, highlights the connection between client experience and whether a woman chooses skilled birthing care or not. Many factors affect perceived and actual quality and accessibility, such as service cost, quality monitoring, and the governance environment for financing and regulation. This discussion will use the concepts of perceived accessibility and quality, as distinct from objectively measured accessibility and quality, as a framework to consider the influence of the social context for the SHE role and its influence on women's uptake of services and gender and power dynamics.

### Perceived accessibility

In Bangladesh and globally, rural areas face a more limited supply of providers and more significant challenges to ensuring high-quality, respectful care among providers [5]. The difficulty in improving provider coverage in underserved areas and the prevalence of disrespectful care is well-documented and persistent [11, 13, 14].

The considerable body of evidence on frontline health workers (FLHWs) demonstrates that fundamental issues like adequate, regular pay and safe working conditions are essential prerequisites to maintaining a successful frontline cadre of health workers [15]. (The term frontline health worker encompasses community skilled birth attendants, midwives, nurses, and physicians) [15]. Recruitment and retention of midwives, nurses, and physicians through financial incentives and other added compensation are common strategies for a geographic redistribution of skilled providers [1, 16]. Unfortunately, these efforts have failed to identify a stable solution to the adequate supply of providers in underserved areas [17].

While additional factors undoubtedly influence the difficulty of attracting providers in remote areas, the inability to earn an adequate, stable income is critical [18–20]. Solutions that rely on unpaid or underpaid lay health workers in the community are not viable [7] and are not sure to improve *perceived* accessibility.

### Perceived quality

The second mediating pathway considered here—perceived quality—is even more complex in its relationship to the uptake of services; the WHO acknowledged in 2014 guidelines on preventing pregnancy-related morbidity and mortality that respectful care still defies definition [21]. Researchers have identified involving women in their care and preparing a supportive environment that supports the woman's choice of companionship as a crucial element of respect [22]. In addition to being a fundamental right, respectful care significantly affects whether and where women seek care [23].

Factors like distance, lack of ancillary services, and desire for a cesarian section affect women's choice to give birth outside a facility. Avoidance of care that does not meet the standards for respectful care is also a significant driver for opting for non-facility deliveries [21, 24]. In response to disrespectful care, women frequently seek care from traditional birth attendants and deliver at home [25].

Simply ensuring an adequate number of providers practicing in underserved areas will not adequately address the challenge of ensuring equitable access to maternity care that is both skilled and respectful [13]. Underlying factors increasing the likelihood of receiving disrespectful maternity care include caste, class, race discrimination, harmful gender norms, and social status. Strategies that incentivize providers from elsewhere to practice in underserved areas may increase the availability of providers. However, they may not increase perceived accessibility or respectfulness of care if newly recruited providers are more urban, of higher social status, or of different ethnic or language groups than their clients, which is likely.

### Approaches to improving perceived quality and accessibility

Approaches to improving quality in ways valued by women are a critical need. For example, an intervention in Afghanistan that prioritized cultural compatibility in underserved areas by working with regional midwifery training centers found high satisfaction among midwives and their clients [27]. They may enhance the attractiveness of the service to individual clients by marrying clinically high-quality care with respectful, culturally congruent care.

An alternative approach must also establish a mechanism to ensure sustainable financing to ensure adequate provider income in underserved areas and facilitate a respectful relationship between providers and clients. One widely employed strategy to address the need to pay FLHW is to rely on a cadre of "volunteer" community-based providers. A risk in designing programming to extend access to health services is that the FLHW/CHW role may shift responsibility, work burden, and even financial contributions onto FLHWs/CHWs as individuals. For example, Schaaf et al. [28] observe that targeted vertical programs relied heavily on volunteer or minimally compensated community health workers to extend the program's reach. Closser and Maes discuss the "appropriation" of the role of the CHW. In these situations, the scope of duties and time commitment demanded of "volunteer" CHWs far exceed the typical expectations of a volunteer role [29]. Over-reliance on these predominantly female, lower-status cadres can decrease their effectiveness and undermine their impact among their social peers in the community as models of women respected and compensated for critical health services.

### Skilled Health Entrepreneurs: a new approach

The Skilled Health Entrepreneurs^1^ (SHE) model emerged from a collaboration between CARE International in Bangladesh, Bangladesh's Ministry of Health, and other partners. This coalition proposed creating a sustainable system to ensure SBA services are available in the remote, underserved rural Sunamganj District in the Sylhet Division, the northeast region of Bangladesh. Skilled providers were scarce in government facilities for at least two significant reasons. The cost of staffing many small clinics in remote locations can pose a substantial obstacle to the health system because of the high per-beneficiary cost for staffing in sparsely populated areas [30]. The government facilities struggled to retain those health workers they successfully recruited in the few rural facilities they could support [30]. Residents were accustomed to seeking delivery care from untrained private birth attendants [31]. The robust market for private traditional birthing care signals a gap in publicly provided services, in quality, quantity, or both. While the care provided by traditional birth attendants might not have met clinical quality standards, it was providing value to clients, potentially through convenience and culturally appropriate, respectful care.

The SHE model proposed increasing the availability of high-quality care and stabilizing access to care from SHEs by selecting residents of the area. As community members, they were less likely to leave the site and more motivated to improve health outcomes for those giving birth in their areas. With support from program staff, they also negotiated a standardized, sliding-scale fee schedule that allows them to continue generating revenue independently while ensuring low-income women can access their services [5].

The program design included measures to increase the capacities of the SHEs in ways beyond the traditional techno-managerial training and supervision in technical skills, such as growing and controlling their earnings and expanding their professional skills. The program intended to support women from the community, as social peers of clients and long-term residents, in becoming recognized, respected health workers linked to the public system while protecting their livelihood and improving quality and access to maternal health services [32] This paper will describe the SHE program's design elements to enhance SHE empowerment in the academic literature on social power and FLHWs.

Hossain, et al. [5] described the Skilled Health Entrepreneur program. The project's purpose was to provide clients with the option of a maternal health service provider that meets clients' needs and preferences. Women in the community preferred traditional birth attendants because they were available outside of business hours, accepted non-monetary payments, and shared social norms and beliefs [5]. The SHE program provided training to fellow community members so that women could receive services from their trusted, culturally congruent providers while ensuring that services offered were safe, high-quality, and linked to referrals for complications.

The project included five central interventions: selection and training of private birth attendants, social entrepreneurship capacity building, community engagement to establish the new cadre in the community, linkages to quality monitoring and referral facilities, and mechanisms to bolster the community's financial support of the program's activities. The program selected participants by inviting applications and conducting interviews and written exams. Women aged 25 to 40 years with at least ten years of schooling were eligible to apply. Over the 5-year life of the project, 319 completed the training.

The project delivered 3 months of training in health service and promotion. The clinical and health promotion training prepared SHEs to support a comprehensive maternal and child package, including antenatal care, assistance in uncomplicated deliveries, postnatal and newborn care, referral for complications, family planning counseling, short-term family planning method provision, and referral. The program used MOH training materials and trainers based on WHO standards. The program also linked SHEs with community support groups, community health workers, government health facilities, and supervisors. See Hossain et al. [5] for more details on the program in general. Once SHEs were prepared to offer services, the program provided ongoing supervision and professional development, including mobile skill labs and advancement opportunities to serve as trainers for incoming new SHEs.

The program also coordinated an alignment between municipal authorities, the health department, and the SHEs. As a result of CARE's coordination, the Health Department provided SHEs with an ongoing supply of health commodities, such as iron folate tablets, soap, and misoprostol, and refresher training. The SHEs charged clients on a sliding scale negotiated by the local government and community representatives. Prices paid were independently monitored periodically. Program staff collaborated with local leaders to explore mechanisms to extend care to the lowest wealth quintile care free of charge.

Over the project's life, SHEs accomplished 47,123 skilled deliveries and dispensed 2.7 million folic acid tablets. As of the end of the program, the median monthly earnings of the SHEs was 5000 BDT (67 USD), compared to 1500 BDT (20 USD) at the beginning of the program. SHEs are formally linked with 136 community clinics and 29 union councils on health and family welfare [33]. A mid-term analysis found that women in the coverage area were more than twice as likely to have delivered with a skilled birth attendant present at their most recent childbirth than at the beginning of the program [5]. The end-line assessment conducted in 2018 demonstrated significant achievements. The percentage of women using a skilled attendant during birth increased from 13.4 to 37.4% in the intervention area compared to 21.4% to 35.8% in a comparison district. Neonatal, infant, and under-five mortality rates all showed similar improvement [33] (see Table 1).

**Table 1:** Change in SHE project outcomes across comparison/intervention areas from baseline to endline

	Comparison area		Intervention area	
	Baseline	Endline	Baseline	Endline
Skilled attendant at birth	21.4%	35.8%	13.4%	37.4%
Neonatal mortality rate	37	29	42	33
Infant mortality rate	60	36	78	46
Under-five mortality rate	76	40	97	52

Sarker et al. (2019)

#### Compensation: financial and marketing skills building

**Fig. 1:**
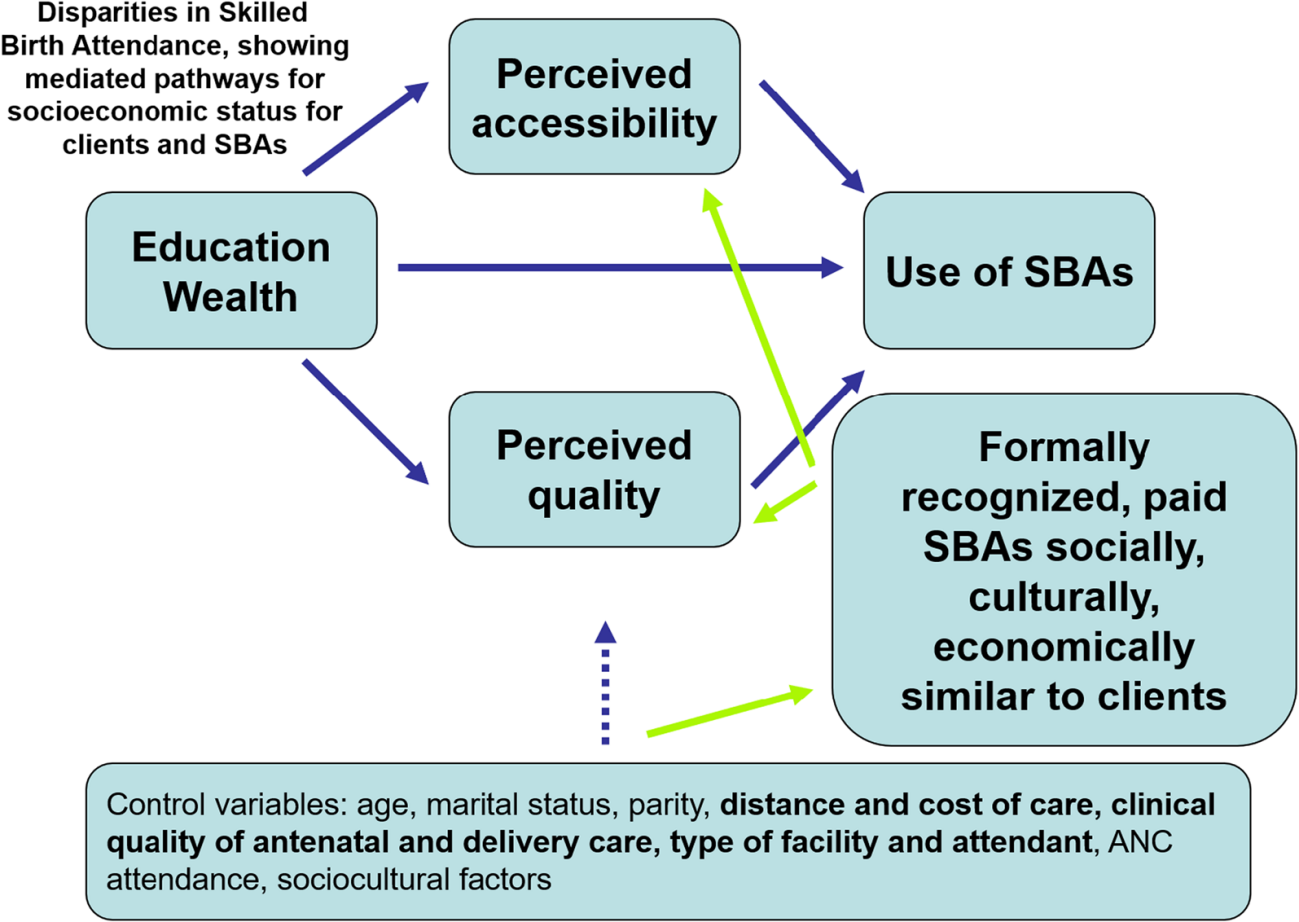
Mediating pathways in uptake of skilled birthing care

One of the intervention arms most directly related to an increase in SHEs' social power focused on building their capacity to earn an adequate income. SHEs developed two potential sources of revenue: direct fee-for-service charges for maternal health services and the sale of health-related products. SHE revenue was not the sole source of household income, however. According to the program’s intake questionnaire administered to SHEs, most SHEs have some additional household income from another adult earner, and some may have had other sources of revenue as individuals unrelated to SHE duties. In addition, SHEs may compete with other providers of similar goods and services. Including income as an element of SHE empowerment should not be considered a comprehensive economic analysis but rather one of the multiple components influencing SHEs' social power (Fig. 1).

The program facilitated a market analysis process with the SHEs. The intervention included a 2-day social entrepreneurship capacity-building workshop drawing on a market analysis of the local market and developing business plans. The workshop covered targeting their service offerings and minimizing conflict with untrained traditional birth attendants. The SHEs received coaching from facilitators skilled in entrepreneurship to determine their potential clients' market size and characteristics. They developed individual business plans targeted to their communities, including outreach to potential clients. The project also conducted promotional and marketing activities ranging from health awareness days to stakeholder meetings to print, video, and media outreach. Another program element connected the SHEs to a supply chain of saleable commodities, such as non-prescription medicine, nutritional supplements, and baby care articles, at wholesale prices. The SHEs then resold these items at a small profit [30].

#### Professional engagement and community recognition

Other project elements contributed to SHEs' agency and external recognition by enhancing their recognition as valuable contributors to the community by authorities outside their homes. CARE's training to the SHEs earned them accreditation by the Bangladesh Nursing Council as a community skilled birth attendant, a professionally recognized designation in Bangladesh [32]. The professional development and skills-building component included coaching by nurses and physicians and organized rotations for the SHEs in healthcare facilities. These inputs conferred legitimacy and status on previously marginalized traditional providers.

Also, the project facilitated negotiation among the SHEs, the local municipal authorities, and the closest primary healthcare facility to establish a formally recognized role for the SHEs. This process set the sliding-scale fee structure discussed above. These negotiations afforded the SHEs recognition as accredited community midwives and secured support from local and neighborhood leaders to provide safe, clean space to perform services and accompaniment on travel to remote locations for home deliveries. The program developed a Memorandum of Understanding between the SHEs and the union parishads and negotiated specific budget line items in UP budgets to supervise the SHEs (These line items did not cover SHE remuneration.)

The formal recognition of their authority and value afforded them greater personal power in negotiating with family and community members about their mobility and control over resources. The provisions for their security removed the threat of violence, stigma, and harassment that could otherwise have accompanied their professional activities.

#### Agency: personal power to act

A third pillar of the program's approach to empowering SHEs was to build their sense of agency on an individual level. Program activities included group planning sessions among SHEs for the SHEs to engage with each other (As each SHE worked in a different neighborhood geographically, the risk of competition among SHEs was minimal). Also, program facilitators worked one-on-one with SHEs, identifying what changes could further develop their businesses [34]. For example, when a regular review revealed that one SHE was not earning as much revenue as targeted, program facilitators examined the factors affecting her ability to make money through her work. They found those factors to include a lack of family support and insecurity when visiting clients in remote locations. The action plan included family support for childcare, introductions to community members, and expanding the products she could sell to generate revenue. In the end, her revenue well exceeded her target [34].

### Limitations

The primary limitation of this discussion is that it is a purely descriptive exercise. A deeper examination of the SHE program provides insight into where and how the SHE approach may be broadly relevant. However, the merit of the approach cannot be demonstrated without empirical data analysis. Further research should cover both the causal pathway and the ultimate outcomes of the model.

Also, context presents a dilemma in this approach. One of the keys to the success of the SHE model was its careful observation of the factors driving women's choices in obtaining birthing care in this setting. The participatory design process allowed for significant tailoring to the market forces and client preferences unique to Sylhet District in rural Bangladesh. Notably, birthing care from traditional birth attendants was in demand before the SHE program and was an essential prerequisite. This demand for birthing care may be necessary for this model to be helpful.

## Discussion

According to Renfrew, et al., any comprehensive solution to introducing and supporting an influential health worker cadre must include minimum educational requirements and processes to ensure training, licensure, and regulation and be systematically integrated into the health system [7]. The SHE program met those criteria and improved birth outcomes. The SHE successfully established a private SBA cadre that enhanced their social power and technical skills in settings challenging to access through the mainstream health system. The SHE model stands out from many adopted globally for this purpose, such as Ethiopia's Women's Development Army and Nepal's Female Community Health Volunteers [29, 35]. The SHE model dedicates concerted efforts to enhance women's decision-making authority, status in their work lives, and economic independence.

Witter (2017) cite concrete measures to address gender barriers as an essential element of building a stable health workforce suited to meet the needs of vulnerable populations [36]. In the SHE program, recognizing the SHEs as sanctioned health service providers legitimizes their status in the community. As community members before receiving SHE training, the SHEs are more likely to be rural, less educated, of marginalized ethnic ups, and lower status than most mainstream service providers. The introduction of peer or near-peer women with well-respected, well-compensated roles among their neighbors may have a powerful effect on other women and offer a model for their lives in different fields.

Focusing on enhancing the SHEs’ agency, voice, and well-being is necessary for this transformative potential. Asking a traditional birth attendant to assume more work for little or no money may increase the burden of unpaid labor on her and also reinforce existing harmful power relations (28). Calling on CHWs to provide services with no guarantee of compensation and refer to facility-based care providers reinforces the notion that it is her feminine duty to care for her neighbors and is more naturally caring and motivated. The SHE model structurally counters those harmful notions. Instead, the SHE model reinforces the perception that the caretaking work, often performed unpaid, usually by women, is worthy of the respect and economic investment of the community.

The importance of class, caste, and race in these power relations also influences the SHE's role. SHEs are more likely to be of lower status on several criteria, such as wealth and education level, than female FLHWs with more training and authority, such as nurses and female physicians [37]. Part of the transformative power of a model like the SHEs is that they are women from the same community and background and have less elite status otherwise. Services offered at the site preferred by the client by a social near-peer coach in prioritizing client-centered care communicates a high value placed on the client's preferences [36].

The sustainability of such approaches is a crucial element of any potential for long-term success or expansion of the SHE model and similar interventions. The fundamental sustainability strategy rests on market forces. The SHEs’ ongoing presence depends on their continued ability to provide services and charge for them. The SHEs could continue earning a substantially increased income from their service provision by the end of the program. The program phased out any direct financial support to the SHEs well before the program concluded. Fundamentally the sustainability strategy is for the SHEs to continue to cost-recover for their services, whether from private clients or through reimbursement from public payers for those unable to pay.

The sustainability of additional support activities remains challenging in at least two ways. First, supervision and entrepreneurship support was provided by grant funding. Supportive supervision and in-service training would require additional approval and investment from health authorities or elsewhere. A combination of health and other agencies at multiple levels (municipal, district, and national) could provide the moderate additional oversight needed to assure quality at a much lower cost than alternatives like providing salary support to community-based SBAs or extending the availability of facility-based SBAs. Secondly, ensuring sustainable financial resources to ensure access to care for lower-income families is a critical challenge for the sustainability of this model. Municipal budgets and community savings groups contributed funding to allow the SHEs to cost-recover services provided to mothers unable to pay during the program. Still, those arrangements were difficult to formalize and vulnerable to changes in budget allocations. Allowing the SHEs to receive reimbursement for skilled delivery services provided outside the facility would be one option for ensuring sustainable financing for SBA services for lower-income clients.

## Conclusion

Taken within the growing body of scholarly work demonstrating the potential benefit of supporting positive gender norms and power dynamics among frontline health workers, these findings suggest some recommendations for health service delivery policy and practice:

- Support robust investment in financing mechanisms to ensure adequate financial compensation for community health workers, especially predominantly or exclusively female cadres.
- Build meaningful commitment to including community-based FLHW cadre in decision-making and planning within the health system through binding agreements among government and private sector stakeholders at local, as well as district and national, levels.
- Include support in addressing gender-related barriers to paid work among female frontline health workers in supervision protocols and intervention design (Such support may include items in the SHE approach like coaching in negotiating social norm barriers among families and training on professional business skills such as public speaking and financial management.)

In addition, an assessment of the SHEs' experience and assessing health outcomes and social relations in the broader community can provide insights into the social role she fills. Understanding the effect of the SHEs’ agency on the women in the communities they serve is vital for the effective implementation of recommendations in other contexts.

Building on these learnings and implementing these recommendations could contribute to expanding women’s access to safe, acceptable care and strengthening social norms supportive of women in influential, professional roles.

## Data Availability

N/A (No datasets were presented in this article.)

## Abbreviations

FLHW: Frontline health workers
SBA: Skilled birth attendant
SHE: Skilled Health Entrepreneurs
UP: Union Parishad

## Footnotes

1 In the first phase of the project the SHEs were known as Private Community Skilled Birth Attendants (PCSBAs) and are mentioned in the cited project documentation interchangeably as SHEs and PCSBAs.
